# Elimination of HIV-1-Infected Primary T Cell Reservoirs in an *In Vitro* Model of Latency

**DOI:** 10.1371/journal.pone.0126917

**Published:** 2015-05-19

**Authors:** Stephen A. Rawlings, Francis Alonzo, Lina Kozhaya, Victor J. Torres, Derya Unutmaz

**Affiliations:** 1 Department of Microbiology, New York University School of Medicine, New York, New York, United States of America; 2 Department of Pathology, New York University School of Medicine, New York, New York, United States of America; 3 Department of Medicine, New York University School of Medicine, New York, New York, United States of America; George Mason University, UNITED STATES

## Abstract

Establishment of long-lived cellular reservoirs of HIV-1 represents a major therapeutic challenge to virus eradication. In this study, we utilized a human primary cell model of HIV-1 latency to evaluate the requirements for efficient virus reactivation from, and the selective elimination of, latently infected human T cells. Ectopic expression of BCL2 supported the replication and spread of R5-tropic HIV-1 in activated CD4^+^ T cells. After IL-2 withdrawal, the HIV-1-infected T cells survived as resting cells for several months. Unexpectedly, these resting T cells continue to produce detectable levels of infectious virus, albeit at a lower frequency than cells maintained in IL-2. In the presence of HIV-1 inhibitors, reactivation of the resting T cells with γc-cytokines and allogeneic dendritic cells completely extinguished HIV-1 infectivity. We also evaluated the ability of the bacterial LukED cytotoxin to target and kill CCR5-expressing cells. After γc-cytokine stimulation, LukED treatment eliminated both HIV-1-infected resting cells and the non-infected CCR5^+^ cells. Importantly, complete clearance of *in vitro* HIV-1 reservoirs by LukED required a lower threshold of cytokine signals relative to HIV-1 inhibitors. Thus, the primary T cell-based HIV-1 latency model could facilitate the development of novel agents and therapeutic strategies that could effectively eradicate HIV-1.

## Introduction

Highly active antiretroviral therapy (HAART) reduces HIV-1 viremia and leads to substantial reductions in HIV-related morbidity and mortality. However, even after prolonged therapy and undetectable viremia, discontinuation or interruption of treatment can cause rapid rebound of HIV-1 and progression to AIDS [1]. This is due to a long-lived reservoir for the virus that takes advantage of the dynamics of immunological memory and does not naturally decay at a rate that could lead to drug independence in a normal lifespan [2, 3]. T cells infected as they transition from an activated to resting state are recognized as a major source of the HIV-1 reservoir [4, 5]. These quiescent, infected T cells are likely protected from cytopathic effects of HIV-1 because of greatly reduced transcription and replication and thus virus production [6]. It has been proposed that elimination of this reservoir could be accomplished by selective activation and induction of cytopathic effects of virus production in these resting T cells in the presence of HAART, thereby preventing HIV-1 spread to new targets [7–9].

Study of the HIV-1 reservoir *in vitro* has been hampered by the low frequency of latently infected cells *in vivo* [10] and the low viability of cultured, resting T cells. BCL2 is a downstream target of the pro-survival signals of the γc-cytokine (IL-2, IL-4, IL-7, and IL-15) family of receptors [11, 12]. Its overexpression in activated T cells enables survival in the absence of IL-2 [13, 14]. IL-2 would otherwise be needed to maintain the cells and sustain a spreading HIV-1 infection [15–17]. A recent study shows that overexpression of BCL2 in primary T cells withdrawn of γc-cytokines can permit return of the cultured T cells to a resting phenotype similar to the resting cells harboring latent HIV-1 in infected individuals [18, 19]. Thus, this *in vitro* model could be useful to study HIV-1 latency in the setting of primary human T cells. As such, we have adapted this experimental approach to establish an *in vitro* HIV-1 reservoir model using replication-competent virus.

Most approaches to eliminating the HIV-1 reservoir rely on induction of virus replication and self-destruction of the infected, resting T cells. Recently, we also assessed an alternative approach of directly killing infected cells and potential targets by using *Staphylococcus aureus* leukotoxin ED (LukED) that binds and kills CCR5-expressing T cells [20]. We showed that treatment of primary CD4^+^ T cell cultures with LukED can prevent the spread of HIV-1 through the timely removal of infected and uninfected CCR5^+^ (target) cells [20]. In this study, we sought to characterize the ability of this toxin to remove latently infected T cells in an *in vitro* model of HIV-1 latency.

We found that T cells ectopically expressing BCL2 supported a replication-competent strain of HIV-1 and could stably harbor the virus for several weeks (>60 days) when forced into a resting state via cytokine withdrawal. Remarkably, a small subset of resting T cells with integrated HIV-1 continued to produce low levels of virus for several weeks *in vitro*. This resting T cell reservoir in *in vitro* T cell cultures could be successfully cleared by reactivation of the cells with γc-cytokine and allogeneic dendritic cell stimulations in the presence of HIV-1 inhibitors. Furthermore, in the setting of a relatively lower strength reactivation signals through γc-cytokines, LukED-mediated depletion of resting, infected cells and CCR5^+^ T cells completely eliminated the HIV-1 reservoir, such that no virus was detected upon subsequent reactivation. These results illustrate the utility of this *in vitro* model of HIV-1 latency and suggest novel mechanisms for targeting and removing cells that harbor latent virus either through strong reactivation or by elimination of targets of the virus.

## Results

### Establishing latent, replication-competent HIV-1 infection of CD4^+^ T cells

**Fig 1:**
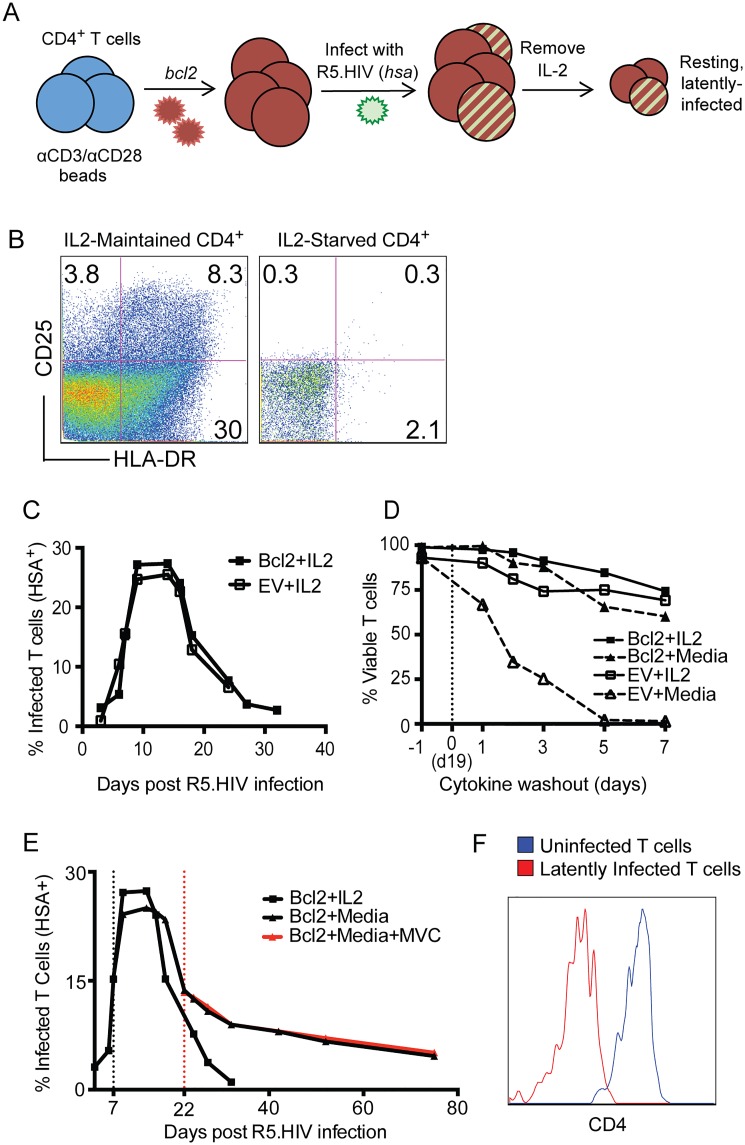
BCL2-transduced CD4^+^ T cells tolerate HIV-1 superinfection and spread and IL-2-withdrawal. **(A)** Schematic of latently infected cell generation showing CD4^**+**^ T cell activation, transduction with BCL2.RFP, superinfection with R5.HIV and subsequent IL-2 withdrawal. Surface staining for HSA (mCD24) expression over time permits tracking of the spread of the virus. **(B)** After activating and transducing primary CD4^**+**^ T cells with BCL2.RFP, cells were starved of IL-2 in their growth medium. FACS analysis of markers of activation of BCL2-transduced, IL-2-maintained (left), and IL-2-starved cells (right). **(C)** Normal spread of R5.HIV infection in T cells transduced with BCL2.RFP (BCL2) or not (EV) and maintained in IL-2 media. **(D)** Viability of T cells as determined by FACS in cultures containing IL-2 or media alone. Cytokine washout occurred 19 days after activation (vertical, dashed line). **(E)** R5.HIV infection in BCL2-transduced cells maintained in IL-2 or not (starved at black, vertical line). Turnover of R5.HIV infection in IL-2-starved cells is also tracked after treatment with maraviroc (MVC; 100ng/mL; begun at red, vertical line). **(F)** CD4 surface staining comparison of uninfected cells (HSA^-^; blue line) and latently infected cells (HSA^**+**^, red line). Representative plots from single donor shown.

In order to create a population of primary CD4^+^ T cells that could be infected with HIV-1 and withstand cytokine withdrawal, we activated total CD4^+^ T cells from healthy, uninfected individuals with αCD3/αCD28 beads and transduced them with a *BCL2*-encoding vector. These cells were expanded for 10–12 days and then superinfected with a CCR5-tropic, replication-competent HIV-1 virus that has murine CD24 (heat stable antigen, HSA) in place of the *vpr* accessory gene of HIV-1 (hereafter, R5.HIV) but has all of the other HIV-1 accessory genes intact [21]. The R5.HIV infection was allowed to spread through the culture for 7–10 days and then IL-2 was removed to induce the cells to enter quiescence (Fig 1A). CD4^+^ T cells that had been transduced with BCL2 and starved of IL-2 for at least 10 days resembled resting, freshly isolated cells in terms of expression of the activation markers CD25 and HLA-DR, compared to activated, transduced T cells maintained in IL-2 (Fig 1B). Infection of T cells by R5.HIV was monitored by staining for expression of the marker HSA on the cell surface. BCL2^+^ cells sustained the spread of R5.HIV in the presence of IL-2 (Fig 1C) and permitted the survival of CD4^+^ T cells in the absence of IL-2. In contrast, cells lacking BCL2 died within 5 days of IL-2 withdrawal (Fig 1D). IL-2 removal from cultures of BCL2^+^ cells that had been superinfected with R5.HIV induced a stabilization of the infected population that could persist for months in the absence of cytokines and was not diminished by the addition of the HIV-1 inhibitor maraviroc (MVC) after latency was established (Fig 1E). Furthermore, latently infected cells both continued to express the marker gene (HSA) and displayed down-regulation of CD4 expression (Fig 1F), in keeping with persistent expression of HIV-1 gene products [22]. Hereafter, “latently infected cells” refers to BCL2^+^ CD4^+^ T cells that were infected with R5.HIV and survived IL-2 withdrawal for at least 10 days.

### Characterizing the HIV-1 reservoir in latently infected cells

**Fig 2:**
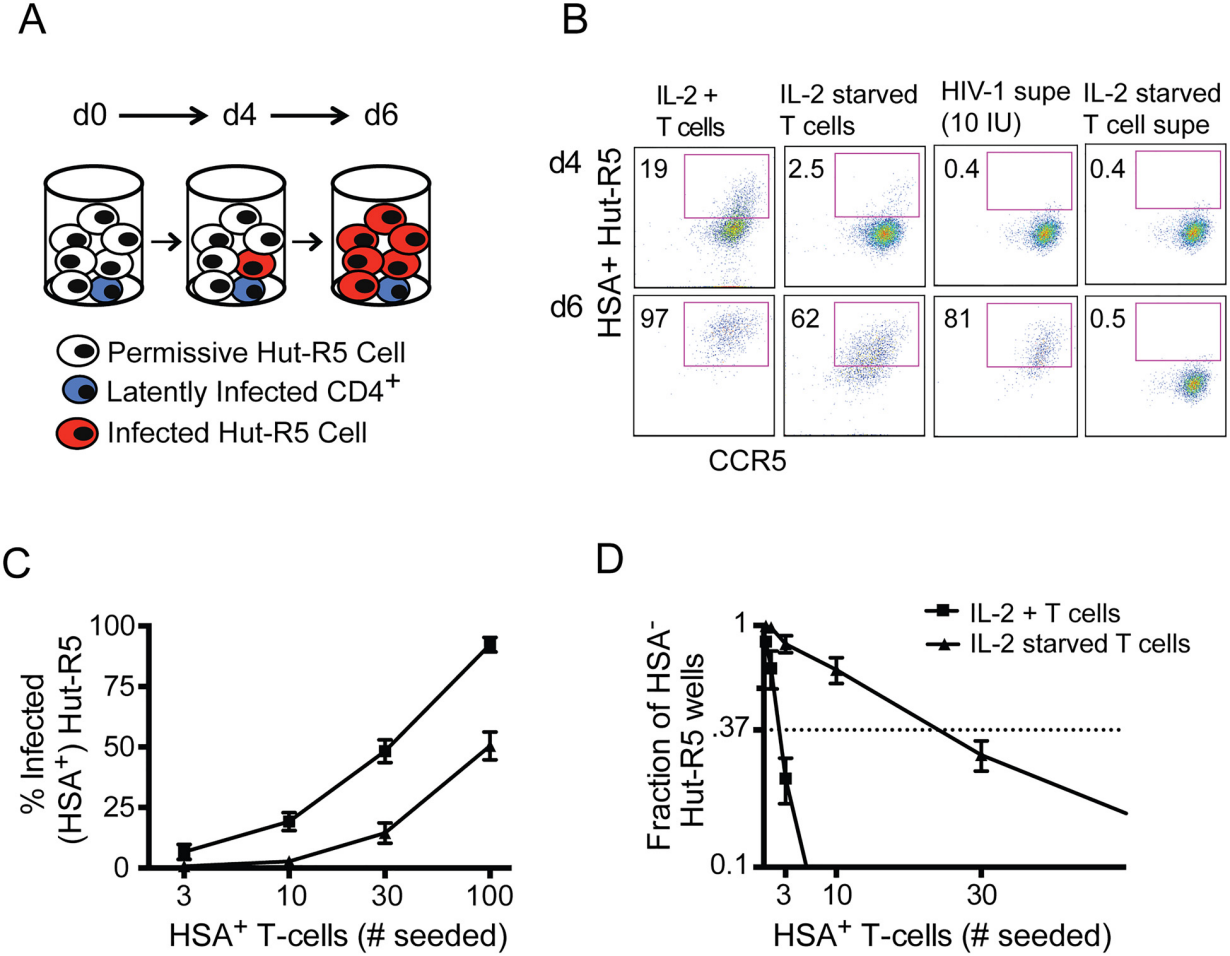
Persistent production of HIV-1 from latently infected T cells. **(A)** Scheme to detect R5.HIV production from latently infected cells via cell-to-cell transmission upon addition to the highly permissive Hut-R5 cell line. **(B)** Determination of infection in Hut-R5 cultures four days (d4; top) or six days (d6; bottom) after co-culture with IL-2-maintained (“IL-2 +”) cells, or latently infected (“IL-2 starved”) cells (10 HSA^**+**^ T cells in each condition). For comparison, cell-free supernatant containing virus (“HIV-1 supe,” 10 i.u.), or supernatant from 10^4^ latently infected cells (“IL-2 starved T cell supe”) was added to the same system to confirm the sensitivity of the system and assay for virus in supernatant from latently infected cells. **(C)** Hut-R5 infection four days after addition of increasing numbers of active or resting cells, or free virus. **(D)** Fraction of replicate wells of Hut-R5 cells determined to be R5.HSA-free after six days. Dashed, horizontal line indicates 37% negative wells. Data are representative of at least three independent experiments (B, C) or the mean (±SEM) of four independent experiments (D).

Having observed that expression of the marker gene was not appreciably lost from latently infected cells over time, we sought to evaluate the persistence of infectious viral particle production from these cells. To assay virus production, we made use of the Hut-R5 cell line, a T-lymphocytic line that is highly permissive to HIV-1 infection and rapidly spreads replication-competent strains. We incubated uninfected Hut-R5 cells with latently infected cells to detect production of virus (Fig 2A). Virus was detected by assaying for HSA expression in the Hut-R5 cells after allowing spread for six days. Infectious HIV-1 production could be detected, even from low numbers of latently infected cells, in the presence of Hut-R5 cells, while supernatant from latently infected cultures did not have appreciable amounts of infectious virus (Fig 2B).

We next assessed the Hut-R5 cell infection at a time-point when infection status is linearly related to the amount of input virus. Indeed, the actively infected cells caused a 3-10-fold increase in infection rates for Hut-R5 cells as compared to the latently infected cells (Fig 2C). In order to more finely determine the frequency of virus-producing cells within the latently infected population, we performed a limiting dilution experiment by decreasing the number of HSA^+^ T cells down to 1 cell per/well in replicate Hut-R5 cultures. Approximately 1 in 2 (±0.3) R5.HIV-infected BCL2^+^ cells, maintained in IL-2, produced infectious HIV-1; whereas infectious virus was detectable in 1 in 24.0 (±3.4) latently infected T cells (Fig 2D), which is about a 10-fold lower frequency compared to cells kept in IL-2.

### Reactivating latently infected cells for viral spread and clearance

A long-standing hypothesis of viral reservoirs in HIV-1 infection postulates that the rapid re-appearance of viremia in infected individuals who cease antiretroviral therapy is due to virus production from reactivated, latently infected T cells that can, in the absence of drug-mediated inhibition, spread to uninfected T cells [5, 10]. Similarly, delivering an activation signal to these latently infected T cells in the presence of HIV-1 inhibitors, could cause death of the HIV-1-harboring cells while preventing infection of bystander cells [1, 8]. Hence, we set out to evaluate both of these hypotheses in our *in vitro* model.

Since the latently infected cells constitute a minority population within resting, uninfected CD4^+^ T cells and possess a replication-competent strain of the virus, we reasoned that delivering an activation stimulus to the whole population would simultaneously activate virus production from the latently infected cells (“reservoir”) and create targets among the uninfected cells as they transition from a resting (generally non-permissive) to activated state [16, 17]. If the activation of virus production from the reservoir induced cytopathic effects on latently infected cells, this could be masked as newly infected cells replace the dying ones. Therefore, we prevented spread of the virus to uninfected cells by adding a potent combination of an HIV-1 entry inhibitor, maraviroc, and an integrase inhibitor, raltegravir (MVC/Ra). This approach also permits evaluation of the extent to which the reservoir can be reduced by various reactivation stimuli.

**Fig 3:**
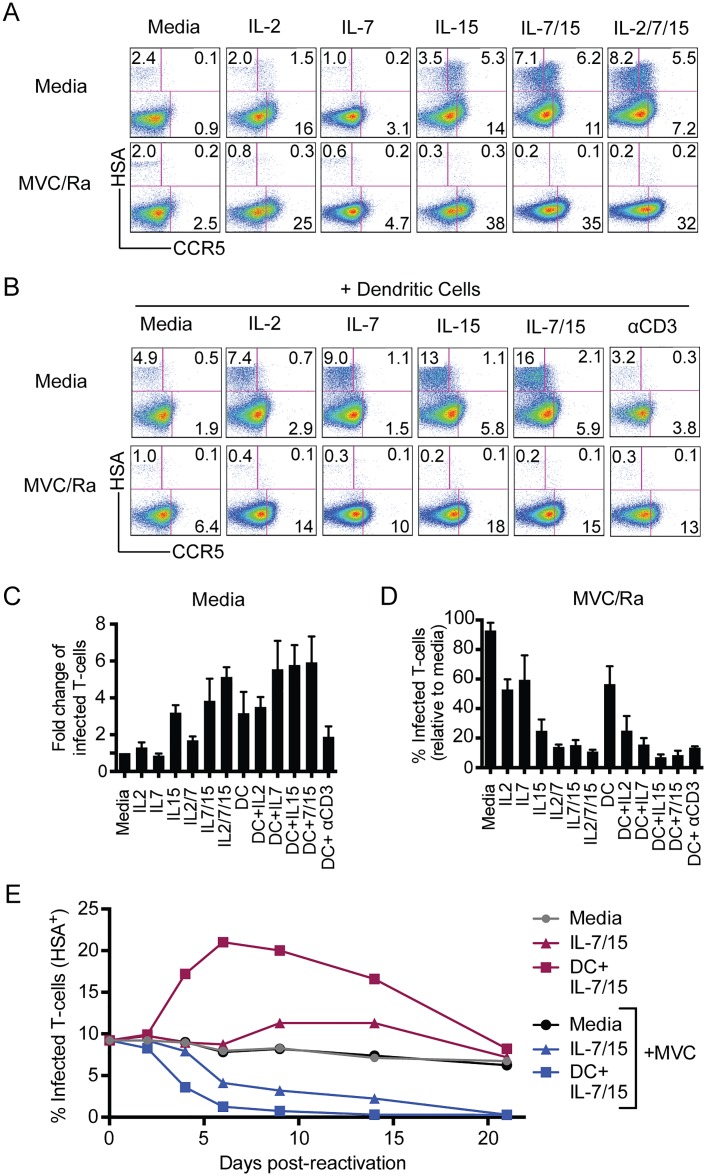
Reactivation and clearance of latently infected T cells. **(A)** Latently infected cells were treated with 20ng/mL of various γc-cytokines alone or in certain combinations in the presence (bottom) or absence (top) of maraviroc and raltegravir (MVC/Ra; 100ng/mL each) and then analyzed by FACS for expression of CCR5 and infection by R5.HIV after eight days. **(B)** Largely the same scheme as (A) but with the addition of allogeneic dendritic cells (DC) at a ratio of 1:5 (DC:T cell). A & B FACS plots from a single, representative donor. **(C)** Fold change in proportion of T cells infected by R5.HIV eight days after treatment of latently infected cells with various reactivating conditions. **(D)** Relative size of the reservoir eight days after reactivating latently infected cells in the presence of HIV-1 inhibitors. C & D: mean ±SEM of three different donors. **(E)** Latently infected, primary T cells were maintained in media or re-activated with γc-cytokines (IL-7/15), or a combination of allogeneic dendritic cells and the same cytokines (DC+IL-7/15) and the change in proportion of infected cells tracked over time. The same conditions were also maintained in maraviroc (MVC). Data are from a single, representative donor.

We used the γc-cytokines IL-2, IL-7, and IL-15 alone or together with monocyte-derived dendritic cells (DC) and in the presence or absence of HIV-1 inhibitors to assess the outcome of different strengths of signals through cytokine receptors (Fig 3A), as well as bystander and T cell receptor (TCR) activation from the DCs (Fig 3B). Combinations of the γc-cytokines resulted in both greater spread, in uninhibited conditions, and clearance of the reservoir when replication was inhibited; and addition of DCs, together with cytokines, both amplified and sped up these responses (Fig 3C–3E).

**Fig 4:**
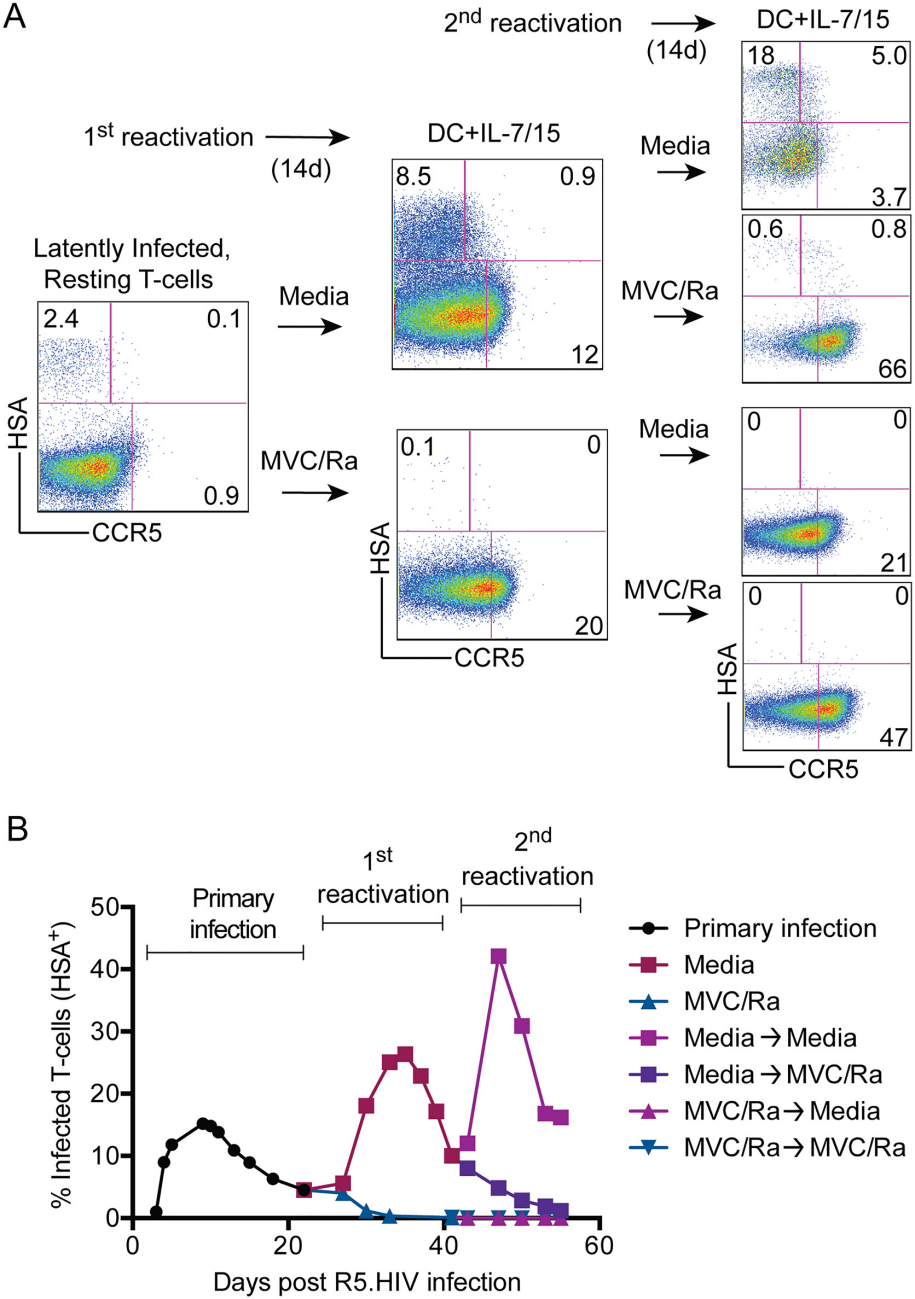
Reduction of the latent reservoirs through activation and inhibition of HIV-1 replication. **(A)** Reactivation scheme for latently infected cells involving DCs, IL-7, and IL-15 in the presence or absence of HIV-1 inhibitors (maraviroc and raltegravir; 100ng/mL each; “MVC/Ra”). After cells were allowed to rest, both conditions were reactivated a second time, again in the presence or absence of HIV-1 inhibitors, and presence of R5.HIV was determined by FACS after 14 days. FACS plots are from a single, representative donor of four independent experiments. **(B)** R5.HIV infection of T cells over time as latency is first established (black circles), and then each subsequent round of reactivation with or without HIV-1 inhibitors. Representative course of infection from single, representative donor.

In summary, our findings suggested that reactivation of latently infected cells with a combination of DCs, IL-7, and IL-15 in the presence of HIV-1 inhibitors could reduce the proportion of HSA^+^ cells by >95%. We next asked whether this decrease was sufficient to extinguish the viral reservoir. Therefore, we conducted serial reactivations of latently infected cells in the presence or absence of HIV-1 inhibitors (Fig 4A). We demonstrated that a single, high-strength reactivation in the presence of HIV-1 inhibitors was sufficient to remove replication-competent spread of R5.HIV in subsequent reactivations (Fig 4A and 4B). Reactivating the HIV-1-“cleared” cultures in the absence of inhibitors did not result in any reappearance of infectious HIV-1 and, more stringently, no virus was detectible (data not shown) when Hut-R5 cells were added to the cultures, as described above (Fig 2A).

### Can the *Staphylococcus aureus* toxin LukED clear HIV reservoirs?

**Fig 5:**
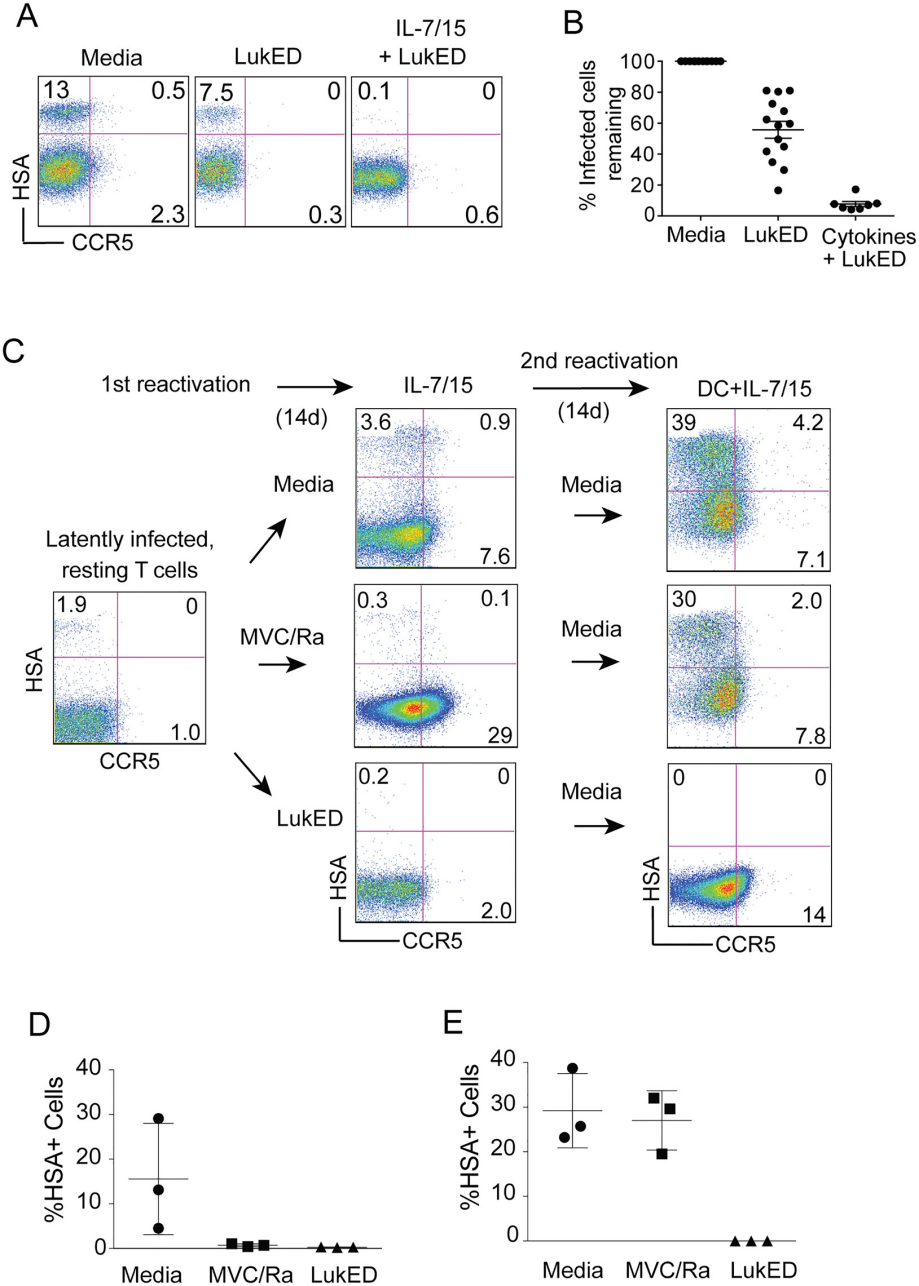
LukED-mediated removal of latently infected and target T cells. **(A)** FACS analysis of latently infected cells from a single, representative donor comparing CCR5 staining and R5.HIV infection after 24-hour treatment with media alone, LukED (10μg/mL); or a 14-day course of cytokine stimulation (IL-7 [3ng/mL], IL-15 [20ng/mL]) and two intoxications with LukED (10μg/mL, day 0 and day 4). **(B)** Comparison of R5.HIV infection remaining after same treatments as in (A). Bars indicate mean infection ±SEM from seven independent experiments. **(C)** Schematic of cytokine stimulation of latently infected cells and concomitant treatment with media alone (top), HIV inhibitors (middle), or two intoxications with LukED as in (A)(bottom) and subsequent R5.HIV infection rate was determined by FACS. After indicated treatment, cells were reactivated with DCs, IL-7, and IL-15 for fourteen days and R5.HIV infection was again determined. FACS plots are from a single, representative donor out of three independent experiments. **(D)** Rates of infection and mean ±SEM 14 days after first treatment from three independent experiments diagramed in (C). **(E)** Rates of infection and mean ±SEM 14 days after second activation from three independent experiments diagramed in (C).

We next asked if we could directly target and kill latently infected cells and potential targets as a means of depleting the latent HIV-1 reservoir. We first tested to what extent LukED could selectively kill latently infected cells, which had to have originally expressed CCR5 at the time of infection. Although we observed that, upon removal of IL-2, CCR5 expression levels on latently infected cells becomes almost undetectable by cell-surface staining (Figs 3A, 3B and 5A, “media”), intoxication with LukED, which only targets CCR5^+^ T cells [20], eliminated a significant portion of latently infected cells (Fig 5A and 5B). The magnitude of the effect varied across donors, but averaged about half of the size of the resting reservoir (Fig 5B).

Human lymphocytes *in vivo are* usually unable to survive in the complete absence of cytokine signals [23, 24], and a portion of resting T cells *ex vivo* express high levels of CCR5, likely induced by γc-cytokines [25]. In the previous experiments we had used the combination of DCs and IL-7+IL-15, which provide a strong signal due to the presence of additional signals produced by DCs, such as TNF and IL-6 that can potentiate γc-cytokine stimulation [16, 26]. However, in this round we first stimulated the latently infected T cells with a lower concentration of IL-7 + IL-15 and then added LukED. This γc-cytokine stimulation and LukED treatment resulted in direct elimination of more than 90% of the latently infected cells compared to cells maintained without LukED (Fig 5B). Fig 5C shows the various treatments and compares the effects of intoxication (bottom row) versus no LukED (top row). Importantly, when the latently infected cells were restimulated with low-dose cytokine in the presence of MVC/Ra, the reservoir was not completely eliminated (Fig 5C, middle row) and upon reactivation with DCs and cytokines, virus spread similar to the condition without the inhibitors (Fig 5C top panel). However, treating the latently infected cells with LukED during the initial low cytokine stimulation resulted in complete depletion of latently infected cells, even after reactivation with strong signals (Fig 5C, bottom panel), which is sufficient to stimulate spread of any residual reservoirs (Fig 5C top and middle panels). The effect was consistent across three donors—both after the first treatment (Fig 5D), and upon reactivation to check for virus (Fig 5E). We attributed this profound effect of LukED to simultaneous elimination of CCR5^+^ latently infected cells, as well as CCR5^+^ uninfected target T cells in these cultures with low-strength cytokine stimulation.

## Discussion

In the present study, we used an *in vitro* model of HIV-1 latency to assess production of infectious virus from latently infected CD4^+^ T cells and approaches to eliminate this viral reservoir. We demonstrate that low amounts of virus production persist from resting, latently infected cells. Importantly, we found that high-strength reactivation of the reservoir, in the presence of HIV-1 inhibitors, could extinguish latently infected cells through virus-mediated killing of the reactivated cells. We also identified a novel approach to eliminate the HIV-1 reservoir by targeting and removing both latently infected and uninfected, CCR5^+^ target cells using the recently identified staphylococcal toxin, CCR5-binding LukED.

To our knowledge, this is the first reported use of replication-competent HIV-1 in a primary CD4^+^ T cell model that employs BCL2-overexpression and IL-2-withdrawal to establish latency. The BCL2-overexpression system is well suited for studying HIV-1 latency because it recapitulates the resting phenotype of *ex vivo* latently infected T cells and allows for generation of large numbers of latently infected, primary T cells [18]. Other reports of the system use modified HIV-1 that lacks nearly all the accessory genes and is not replication-competent [18, 19]. In these models, infected cells that lose expression of the marker gene (destabilized GFP) after IL-2-withdrawal are considered “latent” and are isolated from cells that maintain GFP-expression before further analysis. However, we did not observe considerable loss of marker gene expression in our model and could not elicit significant amounts of virus from HSA^-^CD4^+^ cells sorted out of latently infected cultures (data not shown). Our finding suggests that a major portion of latently infected cells continues to have transcriptional activity to maintain low-level HIV-1 gene expression, as assessed by marker gene expression (i.e., HSA, in the place of *vpr*). Moreover, CD4-expression on these latently infected cells was consistently downregulated, suggesting other viral genes, such as *nef*, are also being transcribed. Indeed, in this replication-competent *in vitro* model, we were able to quantitatively detect persistent, low-level production of virus from latently infected cells. It is possible that the viral production detected from the latent reservoir could indicate a potential source of low-level viremia detected in patients on HAART, in addition to the proposed stochastic activation of latently infected T cells contributing to persistent viremia [8, 27].

Our finding that latently infected cells continue to produce low levels of virus while also continuing to survive prolonged periods in *in vitro* culture strongly suggests that they are avoiding the cytopathic effects of the virus, possibly due to low levels of virus production. Nonetheless, the cytopathic effects of the replication-competent virus in our model become pronounced when latently infected cells are reactivated with various γc-cytokines and/or DCs. By combining γc-cytokine and DC-mediated reactivation signals, maximal reduction of the reservoir could be achieved—provided that new rounds of infection were blocked by HIV-1 inhibitors. Similar approaches to reduce the *in vivo* reservoir with agents that reactivate latently infected cells have been proposed; such as derivatives of prostratin [28], or the γc-cytokine, IL-2 [29–31]. In our model, the combination of IL-7, IL-15, and DC-mediated signaling is sufficient to remove the HIV-1 reservoir and supports the efficacy of using T cell activation to deplete latent HIV-1. Further studies are needed to develop and test this potentially viable strategy for eradiating HIV-1.

We also show that a leukotoxin from *S*. *aureus* was capable of selectively targeting and killing latently infected cells. Although, optimal clearing of latently infected cells with this approach required stimulation with γc-cytokines, at this strength of signal (without additional DCs) LukED was more effective than HIV-1 inhibitors. As such, this approach to deplete the latent reservoir may be considered as a potential alternative to strong, global immune activation in the presence of HIV inhibitors. We recently showed that LukED interacts directly with CCR5 on the surface of T cells as part of a cascade of events that leads to pore-formation and subsequent lysis of CCR5^+^ cells [20]. Despite the seemingly low detection of CCR5 on the surface of latently infected cells by fluorescently labeled antibody, LukED still preferentially targets these cells and thereby reduces the size of the latent reservoir, while also transiently removing uninfected, CCR5^+^ target cells. This scheme is partially similar to the intervention used in the case of the “Berlin Patient” [32], wherein the cells harboring the HIV-1 reservoir are killed, while the potential of the virus to spread to uninfected cells is reduced by depleting CCR5^+^ T cells.

The results of our experiments with this model reveal an incomplete shut-off of viral gene expression and virus production from latently infected cells. Importantly, our findings suggest that not all HIV-1 “latency” is transcriptionally silent since latently infected cells can tolerate low-level virus production in the highly resting state. This notion may also be important in strategies to induce viral transcription, such as with histone deacetylase (HDAC) inhibitors [33–35], since not all cells constituting the reservoir may be transcriptionally quiescent or have virus integrated in silent chromatin regions. Thus, the reactivation induced by HDACi may not be sufficient to induce viral cytopathy in all latently infected cells. In our latency model of primary, resting T cells, the threshold for reactivation able to stimulate sufficient cytopathy appeared to be relatively high, as it required a combination of IL-7, IL-15, and DCs stimulation. In future studies, it will be important to further dissect and determine these compartments of latently infected cells, which may potentially require different combinations of therapeutic approaches to achieve complete elimination of latent HIV reservoir.

Our findings suggest that short cycles of reactivations with cytokines *in vivo* in the presence of HAART and, ideally, an agent to specifically target latently infected cells such as LukED, would permit lower strength reactivation signals and thus reduced side effects in an effort to eliminate the HIV-1 reservoir in infected individuals. These novel approaches to eradicate latently infected cells may have implications in developing therapeutic strategies to eliminate the residual HIV-1 reservoir in infected individuals.

## Materials and Methods

### Cell purifications and culture

Blood samples were obtained from anonymous healthy donors as buffy coats (New York Blood Center). New York Blood Center obtains written informed consent from all participants involved in the study. Because all the samples were sent as anonymous, the Institutional Review Board at New York University medical center determined that our study was exempt from further ethics approval requirement. Peripheral Blood Mononuclear cells (PBMC) were isolated with Ficoll-Hypaque (Amersham Pharmacia). CD4^+^ T cells were isolated from PBMC using magnetic-bead sorting (Invitrogen, Dynabeads). Purified CD4^+^ T cells were activated using anti-CD3/CD28 coated beads (Dynabeads, Invitrogen) and cultured in RPMI media (Mediatech) with 10% Fetal Bovine Serum (FBS; Atlanta Biologicals) and supplemented with IL-2 (200 U/ml), 100 U/mL penicillin and 0.1 mg/mL streptomycin (P/S; Mediatech); and MEM Nonessential amino acids (Mediatech), Sodium Pyruvate (Mediatech), MEM Vitamins (Mediatech), and GlutaMAX (Invitrogen) at manufacturer’s specified concentrations. IL-7 and IL-15 were obtained from R&D Systems and reconstituted per manufacturer’s instructions. Monocyte-derived dendritic cells (DCs) from healthy donors were generated from CD14^+^ cells as previously described [36]. Briefly, monocyte (CD14^+^) cells were isolated from PBMCs using anti-CD14 antibody-coated bead-based sorting via AutoMACS (Miltenyi Biotec) and were typically more than 99% pure. DCs were generated from CD14^+^ cells by supplementing the culture medium with human granulocyte macrophage colony-stimulating factor (50 ng/mL) and IL-4 (40 ng/mL). Cells were cultured for 3–5 days in the differentiation condition before being mixed with latent cells at a ratio of 1:5 (DC:CD4^+^ T cell).

### HIV-1 inhibitors and LukED

The following HIV-1 inhibitors were obtained from the AIDS Research and Reference Reagent Program, Division of AIDS, NIAID, NIH: Maraviroc (Cat# 11580); Raltegravir (Cat# 11680) from Merck & Company, Inc. Stock solutions were made following recommendations from the accompanying data sheets for suggested concentrations and solvents. Aliquots of each stock were stored at -20°C or -80°C, per recommendations, and fresh aliquots were used for each experiment. LukE and LukD were prepared as previously described [37]. Purified protein eluates were diluted with RPMI and aliquots of this stock solution were stored at -80°C until needed. Fresh aliquots of each subunit were thawed and mixed 1:1 for individual experiments.

### Staining with antibodies and FACS analysis

Cells were stained in complete RPMI media or PBS + 2% FBS and 0.1% sodium azide (FACS Buffer) for 30 minutes at 4°C and washed before running on BD LSR-II flow cytometer. Stainings that included antibodies for CCR5 were performed at room temperature. Cell sorting was done using BD FACS Aria (BD Biosciences). Data was analyzed using FlowJo software (Treestar) and gated on live cells based on forward and side scatter properties, as well as fixable viability dye eFluor 450 (eBioscience). The following antibodies were used to stain cells for FACS: HSA-FITC, CD4-APC, CCR5-APC-Cy7 (BD Pharmigen), CD25-Alexa700, CD69-PacificBlue, HLA-DR-APC-Cy7 (Biolegend).

### Limiting dilution of infected cells

The frequency of HSA^+^, latently infected cells was determined in the T cell culture immediately before each co-incubation study. Three 96-well plates were then seeded with the appropriate number of T cells to contain the following numbers of HSA^+^, latently infected cells per well: 300 (10 wells, 2 wells empty as control), 100 (12 wells), 30 (12 wells), 10 (24 wells), 3 (60 wells), 1 (72 wells), 0.3 (96 wells). 10^5^ Hut-R5 cells were then added to each well and the plates incubated for four days. At this time, 2/3 of the culture was removed and stained for HSA and CCR5 (as staining control), fresh RPMI was added to allow any infection to continue to spread. Six days after the co-incubation was begun, all cells were removed and stained for HSA and CCR5. Wells were scored as positive if >2% of the CCR5^hi^ population was HSA^+^.
